# Quantification of All‐*Trans* Retinoic Acid and Cytokine Levels After Fungal, Viral and Bacterial Infections in the Lung

**DOI:** 10.1111/jcmm.70391

**Published:** 2025-03-03

**Authors:** Samuel A. Krug, Ravineel Singh, Jianshi Yu, William T. Witt, Nageswara R. Pilli, Angela Wilks, Mariette Barbier, Keven M. Robinson, Maureen A. Kane

**Affiliations:** ^1^ Department of Pharmaceutical Sciences, School of Pharmacy University of Maryland Baltimore Maryland USA; ^2^ Department of Medicine, School of Medicine University of Pittsburgh Pittsburgh Pennsylvania USA; ^3^ Vaccine Development Center, West Virgina University Health Sciences Center Morgantown West Virginia USA

**Keywords:** *Aspergillus fumigatus*, cytokine, Influenza A, lung, mass spectrometry, methicillin‐resistant *Staphylococcus aureus*, *Pseudomonas aeruginosa*, retinoic acid

## Abstract

All‐*trans* retinoic acid (atRA) plays a critical role in tissue homeostasis as a master regulator of cellular proliferation, apoptosis and differentiation as well as in immune cell differentiation and function. An active metabolite of vitamin A, atRA has been reported to be reduced in a number of inflammatory conditions in both the lung and gut. Decreases in atRA have been reported in gastrointestinal tissue in inflammatory bowel diseases, radiation‐induced gastrointestinal injury and viral infection. In the lung, atRA is reduced in inflammatory conditions including allergic asthma and radiation‐induced lung injury; however, the impact of infection on lung atRA is not well defined. In this short communication, we quantified atRA and cytokine levels in the lung after fungal, viral and bacterial infections in mice and determined the correlation between atRA and cytokine levels in the lung. atRA was quantified by LC‐MRM^3^, and seven different inflammatory cytokines were quantified by multiplexed immunoassay in mouse lung challenged with Influenza A, *Aspergillus fumigatus* , *Pseudomonas aeruginosa* or methicillin‐resistant *Staphylococcus aureus* . Combined infections were also investigated. Our results show that there is a significant decrease in atRA after infection regardless of infection type. We show an inverse correlation between the decrease in atRA and the increase in inflammatory cytokines IL‐1β, IL‐6, IL‐10 and IL‐12 in lung tissue during infection. Elucidation of the homeostatic regulation of active metabolite atRA is important to understanding disease pathology and may enable future drug development to combat the effects of inflammation and infection.

## 1 Introduction

**FIGURE 1:**
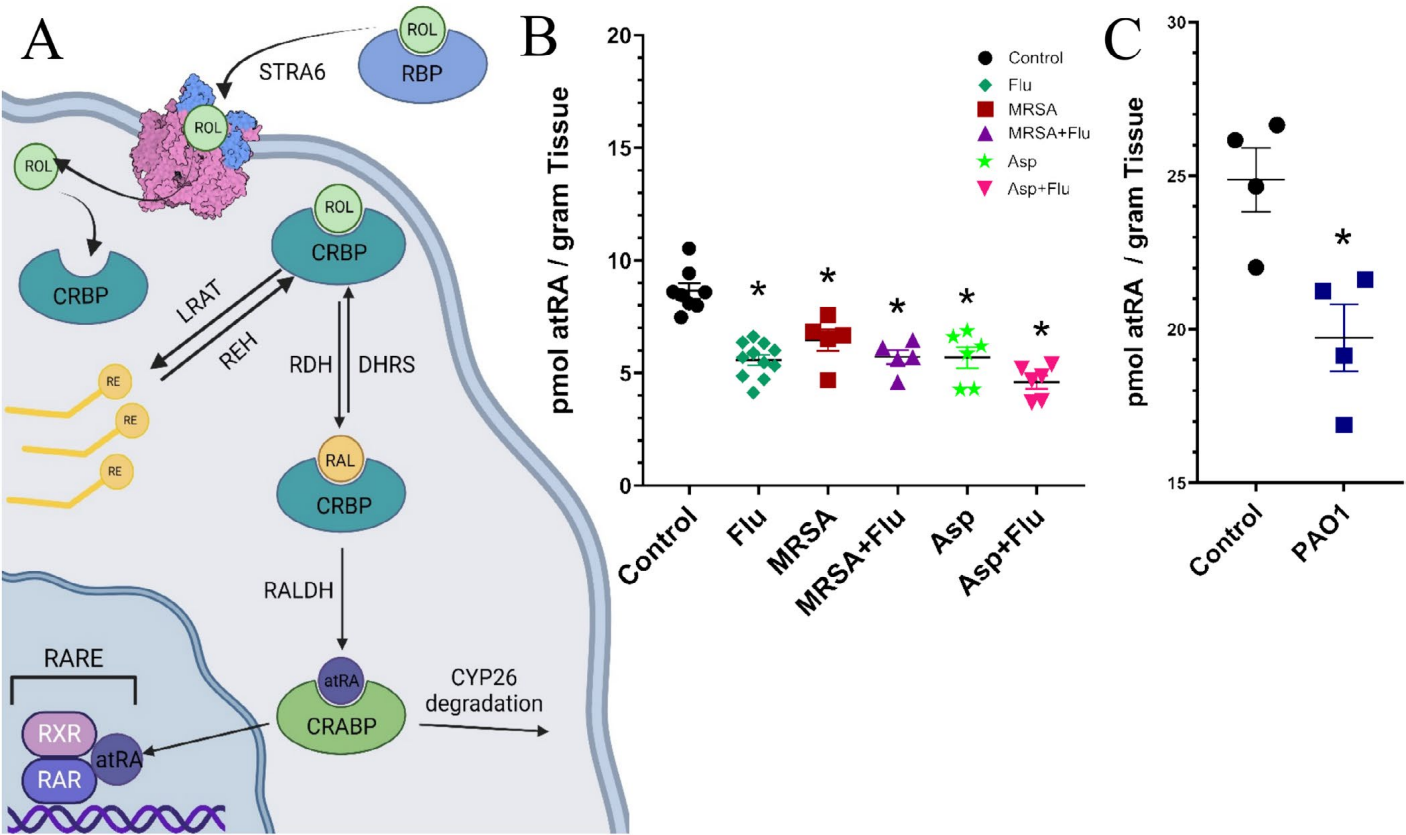
atRA is reduced in lung tissue after viral, bacterial or fungal infection. (A) Biosynthetic pathway of active metabolite atRA. Vitamin A (retinol [ROL]) precursors are obtained from vegetables and fruits in the form of pro‐vitamin A carotenoids or animal sources in the form of preformed vitamin A (ROL, retinyl esters). Vitamin A can be stored in the liver as retinyl esters and mobilised as ROL for transport throughout the body for different cellular functions accompanied by chaperone retinol‐binding protein (RBP). RBP‐bound ROL is imported into the cell by transporter STRA6. Once in the cell, ROL can either be esterified by LRAT to be stored as retinyl esters, or oxidised through two enzymatic steps via retinol dehydrogenases and retinal dehydogenases to convert ROL to retinal (RAL) and subsequently RAL to atRA, respectively. atRA regulates the transcription of hundreds of genes through nuclear receptors, retinoid acid receptor (RAR) and retinoic x receptor (RXR). (B) LC‐MRM^3^ quantitation of atRA from lung tissue for male C56BL/6 mice challenged with *Influenza A* (Flu, *n* = 11), methicillin‐resistant *Staphylococcus aureus* (MRSA, *n* = 5), *Aspergillus fumigatus* (Asp, *n* = 6) or co‐infection (Asp + Flu, *n* = 6; MRSA + Flu, *n* = 5) compared with vehicle control (*n* = 8). C. LC‐MRM^3^ quantitation of atRA from lung tissue homogenate for male CD1 mice challenged with *Pseudomonas aeruginosa* reference strain (PAO1, *n* = 4) compared to vehicle control (*n* = 4). Significance of *p* < 0.05 as compared to control is indicated by “*” according to Student's *t*‐test (unpaired, two‐tailed).

All‐*trans* retinoic acid (atRA) plays a critical role in tissue homeostasis as a master regulator of cellular proliferation, apoptosis and differentiation as well as in immune cell differentiation and function [1, 2]. atRA plays numerous roles in both the innate and adaptive immune response, including regulation of dendritic cell differentiation and function, Treg conversion, innate lymphoid cell (ILC) development and function, and macrophage identity and differentiation during infection [2, 3, 4, 5]. The process of converting diet‐derived nutrient Vitamin A (retinol, ROL) into the active metabolite atRA is highly regulated to ensure precise concentrations of this potent signalling molecule (Figure 1A) [1, 6].

Gut‐ and bronchus‐associated lymphoid tissue have functional and morphological similarities and share some similarities in immune response [7]. Mucosal damage in the small intestine, including inflammatory bowel diseases (IBD, e.g., Crohn's disease and ulcerative colitis), radiation‐induced injury and viral (HIV/SIV) infection, induces a local state of reduced active metabolite atRA in various human, nonhuman primate and mouse models. Limited atRA availability inhibits the number and function of cell populations important to gut homeostasis and immune response that rely on the atRA signal [8]. During inflammatory conditions in the lung, such as allergic asthma and radiation‐induced lung injury, atRA is reduced in human, nonhuman primate and/or mouse [9, 10]. Deficiency of vitamin A is correlated with reduced airway function, can exacerbate allergic asthma [11, 12, 13, 14] and is associated with pulmonary fibrosis [15] in human and rodent models. Therapeutic modulation of atRA impacts inflammatory signals and cell morphology, attenuates radiation‐induced fibrosis and inhibits cytokine production in the lung in human and mouse [16, 17, 18]. However, gaps in understanding how infection may modulate the homeostasis of the retinoid pathway and impact local availability of atRA in the lung are not completely understood. Here, we sought to determine: is atRA altered in the lung after infection; does the impact on atRA depend on lung infection type; and does a change in atRA correlate with select inflammatory cytokines?

## 2 Materials and Methods

### 2.1 Sample Size

Power analysis was used to determine cohort sizes where mouse numbers were justified based on our preliminary data, previous data and literature showing a reduction in atRA levels induced by infection. We used the standard statistical criteria of a power of 0.8, a *p* value of 0.05 and a *K* = 7.85 in all calculations according to the following formula: *n* (number of subjects needed) = *K* (2*σ*2)/Δ2, where *σ* = standard deviation (SD) and Δ = expected difference.

### 2.2 Tissue Harvest

All tissue was flash frozen and stored at –80°C until homogenization for analysis.

### 2.3 Influenza A and *Aspergillus fumigatus* Individual Infections and Co‐Infection

Influenza A/PR/8/34 H1N1 was used to inoculate male C57BL/6 mice at 2000 PFU by oropharyngeal aspiration and then tissue was harvested at Day 8 post‐infection, as previously described [19]. *A. fumigatus* (ATTC 42202) was used to inoculate male C57BL/6 mice at 2.5 × 10^7^ conidia and tissue was harvested after 48 h. For co‐infection, mice were first inoculated with influenza and then challenged with *A. fumigatus* at Day 6 post‐influenza and harvested 48 h after co‐infection. Mice were grouped as control (*n* = 6, no infection), *Aspergillus* only infection (*n* = 6), influenza only infection (*n* = 6) and co‐infection (*n* = 6).

### 2.4 Influenza A and Methicillin‐Resistant *Staphylococcus aureus* Individual Infections and Co‐Infection

Influenza A/PR/8/34 H1N1 was used to inoculate male C57BL/6 mice at 2000 PFU by oropharyngeal aspiration, and then tissue was harvested a week later, as previously described [19]. Male C57BL/6 mice were inoculated with 5 × 10^7^ CFU MRSA USA300, and tissue was harvested 24 h after infection, as previously described [20]. For co‐infection, mice were first inoculated with influenza and then challenged with MRSA at Day 6 post‐influenza, and tissue was harvested 24 h after MRSA infection. Mice were grouped as control (*n* = 5, no infection), MRSA only (*n* = 5), influenza only (*n* = 5) and co‐infection (*n* = 5).

### 2.5 *Pseudomonas aeruginosa* Infection

Male ICR‐CD1 mice were inoculated with 1 × 10^7^ CFU of *P. aeruginosa* intratracheally, as described previously [21]. Tissue was harvested after 24 h (*n* = 4 control, *n* = 4 PAO1 infection).

### 2.6 Retinoid Extraction and Analysis

We used validated methodology for atRA analysis [22, 23]. Tissue (30–100 mg) was weighed and homogenised in 1 mL of saline (0.9% NaCl) by hand in glass. Extraction was performed under yellow lights using a two‐step liquid–liquid extraction with 4,4‐dimethyl‐RA as an internal standard, as previously described [23]. Concentrations of atRA were determined using liquid chromatography‐multistage‐tandem mass spectrometry (LC‐MRM^3^) using atmospheric pressure chemical ionisation in positive‐ion mode with a 6500+ QTRAP hybrid tandem quadruple mass spectrometer (AB Sciex, Foster City, CA) [22].

### 2.7 Cytokine Analysis

Lung tissue was homogenised using Precellys using soft tissue CK14 homogenization beads (Bertin Technologies, France). PBS was added to the tissue in a 1:3/tissue:volume ratio. Lung homogenate was analysed for IL1‐beta, TNF‐alpha, IL‐6, IL‐10, IL‐12, IL‐21 and IL‐22 using a Luminex bead‐based immunoassay analysis (Luminex, Austin, TX) at the University of Maryland Cytokine Core Laboratory.

### 2.8 Protein Quantitation

Total protein from lung homogenate was quantified using the ThermoFisher Pierce BCA Protein Assay kit (Catalogue #23225).

### 2.9 Statistical Analysis

All data is expressed as mean ± SEM. One‐way ANOVA with Bonferroni adjustment was used to compare the control group to the infection groups individually. Control groups and Influenza only groups for both MRSA and *Aspergillus* cohorts did not have significantly different levels of atRA. An adjusted *p* value of < 0.05 was considered significant. Simple linear regression was used to calculate Pearson's *R* and *p* value of cytokine correlation. Statistical analysis was performed using GraphPad Prism version 10.2.0.

## 3 Results

### 3.1 atRA Is Reduced in Lung Tissue After Viral, Bacterial and Fungal Infections

atRA was quantified in lung tissue using a validated and extensively applied LC‐MRM^3^ assay [22]. Figure 1B shows atRA levels quantified in the lung of male C57BL/6 mice after challenge with viral (*Influenza A*; Flu), Gram‐positive bacterial (methicillin‐resistant *S. aureus* ; MRSA) or fungal ( *A. fumigatus* ; Asp) infection as well as combined superinfections of MRSA + Flu and Asp + Flu. Figure 1C shows atRA levels quantified in the lung of male ICR‐CD1 mice after challenge with gram‐negative bacterial ( *P. aeruginosa* , *PAO1*; PAO1). Due to variations in basal retinoid levels in different strains of mice, PAO1 infection was plotted separately from other infections [24]. Figure 1B,C demonstrate that atRA is significantly reduced during infection, ranging from 21% reduced in PAO1 to 49% reduced in Asp + Flu. A similar extent of reductions in atRA was observed in viral (Influenza A), bacterial (MRSA, Gram‐positive; *P. aeruginosa* , Gram‐negative) as well as fungal ( *A. fumigatus* ) infections. Co‐infection did not significantly reduce atRA further when compared to individual infection. These results indicate that infection caused a local atRA decrease in the lung and that decrease was similar after infection regardless of infection type.

### 3.2 Inflammatory Cytokines Increase With Decreasing atRA Concentration

**FIGURE 2:**
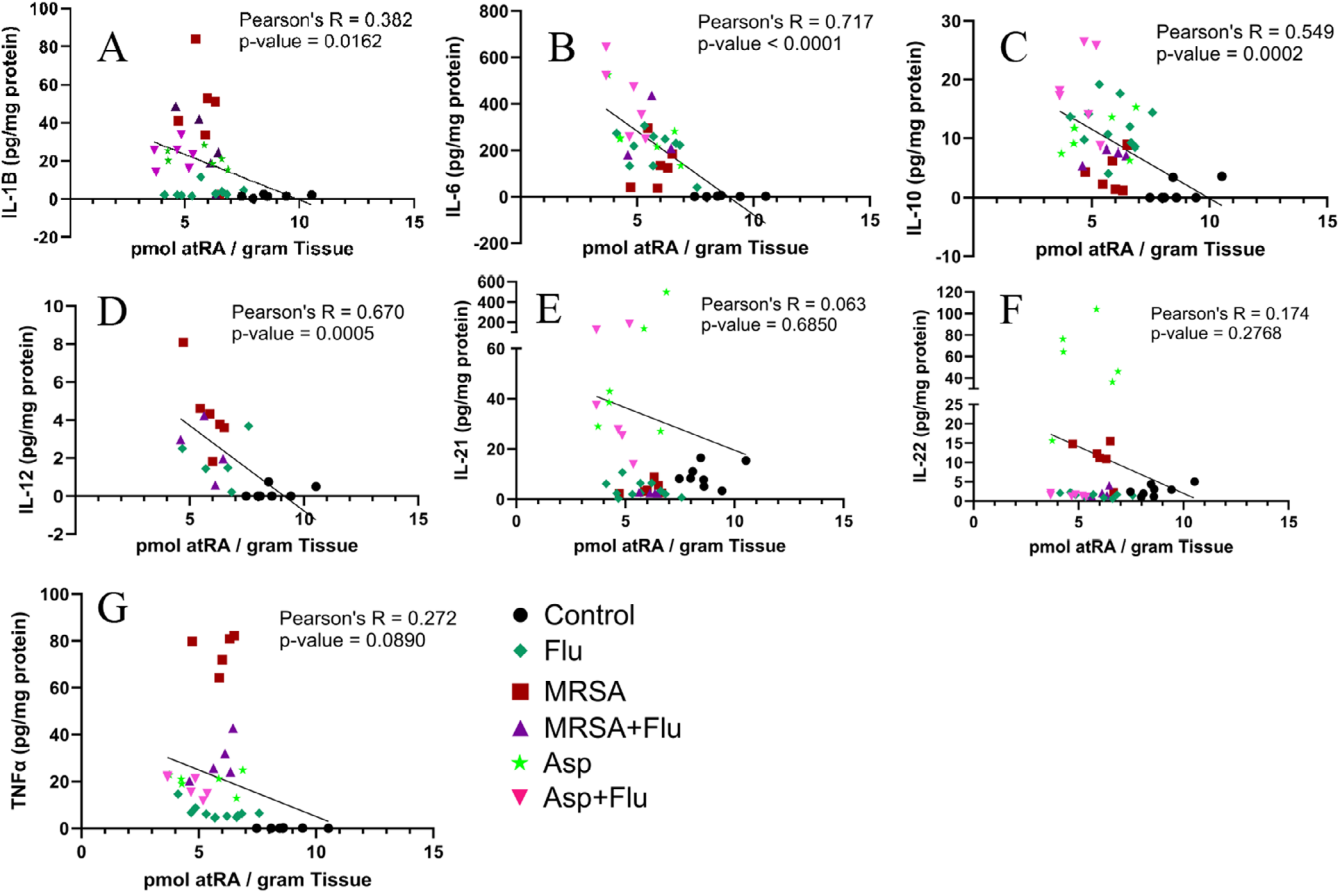
Correlation of cytokine levels with atRA concentration in the lung. Correlation of atRA levels with: (A) IL‐1β, (B) IL‐6, (C) IL‐10, (D) IL‐12, (E) IL‐21, (F) IL‐22 and (G) TNFα. Pearson's *R* and *p* value for linear correlation are provided with each graph. atRA in lung was quantified by LC‐MRM^3^ and cytokines were quantified by multiplexed immunoassay in male C57BL/6 mice challenged with *Influenza A* (Flu, *n* = 11), methicillin‐resistant *Staphylococcus aureus* (MRSA, *n* = 5), *Aspergillus fumigatus* (Asp, *n* = 6), co‐infection (Asp + Flu, *n* = 6; MRSA + Flu, *n* = 5) or were vehicle control (*n* = 8).

Cytokines were quantified in the lung with a multiplexed immunoassay. Figure 2 shows the correlation of cytokine levels in lung tissue as a function of atRA lung concentration for IL‐1β, IL‐6, IL‐10, IL‐12, IL‐21, IL‐22 and TNFα. atRA data from Figure 1B were assessed for correlation with each cytokine using Pearson's *R*. Significant non‐zero slope (*p* < 0.05) was found for IL‐1β, IL‐6, IL‐10 and IL‐12. Pearson's *R* ranged from 0.382 (IL‐1β) to 0.717 (IL‐6). The Asp and Flu + Asp groups are not included in the IL‐12 correlation due to an IL‐12 quantitative value lower than the limit of detection. IL‐21, IL‐22 and TNFα did not have significant non‐zero slopes and had the lowest Pearson's *R* correlations ranging from 0.063 (IL‐21) to 0.272 (TNFα). The low correlations were influenced by Asp infection (lime green star) showing distinctly higher levels for IL‐21 and IL‐22 compared to other types of infection, and MRSA infection (red square) showing distinctly higher levels of TNFα as compared to other infection types. These data show that lung atRA correlated inversely with lung IL‐1β, IL‐6, IL‐10 and IL‐12 with decreases in atRA correlating with increases in cytokines after infection.

## 4 Discussion

Here, we have shown that atRA decreased in lung infection, regardless of infection type, after viral, fungal or bacterial infection (both Gram‐positive and Gram‐negative bacteria). This is consistent with previous studies that have shown atRA to be decreased in the gut after viral infection and decreased in the lung during inflammatory diseases [9, 25]. We additionally show that atRA correlates with some inflammatory cytokines across infection types, including IL‐1β, IL‐6, IL‐10 and IL‐12. Other cytokines assayed had weak correlation with atRA due to distinctly different cytokine levels in either Asp (IL‐21, IL‐22) or MRSA (TNFα) infection. atRA has been shown to regulate IL‐1β, IL‐6, IL‐10 and IL‐12 in various immune cells [2, 26, 27]. IL‐6 had the highest correlation with atRA levels in the lung. In models of radiation‐induced pulmonary fibrosis, atRA inhibited IL‐6 production through a protein kinase C (PKC)‐δ/NF‐κB–mediated mechanism [18].

Local changes in retinoid homeostasis that result in a decrease in atRA levels could have a number of important effects in the lung. As gut‐ and bronchus‐associated lymphoid tissues have functional and morphological similarities [7], previous investigation regarding the role of atRA in modulating intestinal innate immunity in health and inflammation‐associated disorders may provide common mechanisms for future study in the lung [2]. This single RA nutrient can critically impact cell‐mediated immunity and gut cell homeostasis [28]. For example, after mucosal injury in the small intestine, RA provides a differentiation prompt that controls the fate commitment of opposing pre‐DC derived lineages [28]. Innate lymphoid cells (ILCs) are important to tissue homeostasis, repair and host defence in the mucosa of the gut and lung that are most exposed to the environment [29]. RA promotes ILC plasticity, homing of ILC1 and ILC3 to the intestine, and may also be an important microenvironmental signal for ILC migration to and within lung tissue [29]. In tissues damaged by infection, inflammatory diseases and irradiation, ILCs contribute to repair likely due to their local migration and accumulation to the local sites of inflammation within the damaged tissue [29]. atRA also plays a role in stimulating mucus secretion to maintain a physical barrier for the host from the environment [30]. M2 macrophages have been previously shown to produce more RA and M2 macrophage atRA biosynthesis capacity correlated with tissue levels [31]. Some responses may depend on both atRA and cytokines. For example, pro‐inflammatory cytokines in conjunction with atRA promote dendritic cell activation and effector T cell production [5]. M1 and M2 macrophage differentiation during infection is driven by atRA in combination with plasma factors and cytokines [4].

A fundamental understanding of the homeostatic mechanisms of key signalling molecules, like atRA, and their disruption in various infection types may advance the rational development of new therapeutics. Elucidating the role of endogenous retinoids in the pathogenesis of infection will aid in future retinoid therapeutic development. With the rise of antibiotic‐resistant pathogens, synthetic retinoids have been explored as a potential therapeutic strategy. atRA has demonstrated efficacy in treating *Pneumocystis* pneumonia, *Mycobacterium tuberculosis* , *S. aureus* and *A. fumigatus* [32, 33, 34, 35]. Additional studies have examined the potential for synthetic retinoids to combat infections from *P. aeruginosa* and MRSA [36, 37]. In addition to anti‐bacterial and anti‐fungal activity, previous histological examinations in inflammation models revealed that the inflammatory phenotype can be mitigated by retinoid treatment [38]. In conclusion, these data show quantitation of endogenous atRA, a potent signalling molecule, in lung tissue after various infection types and its correlation with indices of inflammation, which provides key information that will aid in the design of future studies to investigate both the mechanism of disease and novel drug development.

## Author Contributions

**Samuel A. Krug:** conceptualization (lead), formal analysis (lead), investigation (lead), visualization (lead), writing – original draft (equal), writing – review and editing (equal). **Ravineel Singh:** investigation (supporting), writing – review and editing (supporting). **Jianshi Yu:** investigation (supporting), writing – review and editing (supporting). **William T. Witt:** investigation (supporting), writing – review and editing (supporting). **Nageswara R. Pilli:** investigation (supporting), writing – review and editing (supporting). **Angela Wilks:** funding acquisition (equal), resources (equal), writing – review and editing (equal). **Mariette Barbier:** resources (equal), writing – review and editing (equal). **Keven M. Robinson:** conceptualization (equal), funding acquisition (equal), resources (equal), writing – review and editing (equal). **Maureen A. Kane:** conceptualization (lead), formal analysis (equal), funding acquisition (equal), project administration (lead), resources (equal), visualization (equal), writing – original draft (equal), writing – review and editing (lead).

## Ethics Statement

Animal experiments were performed in accordance with the National Institutes of Health Guide for the care and use of laboratory animals. The protocols used were approved by either the University of Pittsburgh or the West Virginia University Animal Care and Use Committee.

## Conflicts of Interest

The authors declare no conflicts of interest.

## Data Availability

The data that support the findings of the study are available from the corresponding author upon reasonable request.
